# Adjuvant Iodine-125 Brachytherapy for Hepatocellular Carcinoma after Complete Hepatectomy: A Randomized Controlled Trial

**DOI:** 10.1371/journal.pone.0057397

**Published:** 2013-02-28

**Authors:** Kaiyun Chen, Yong Xia, Hanning Wang, Fanglian Xiao, Guoan Xiang, Feng Shen

**Affiliations:** 1 Department of General Surgery, the Second Provincial People’s Hospital of Guangdong Province, Guangzhou, P. R. China; 2 Department of Hepatic Surgery, Eastern Hepatobiliary Surgery Hospital, Second Military Medical University, Shanghai, P. R. China; Northwestern University Feinberg School of Medicine, United States of America

## Abstract

**Background:**

Tumor recurrence is a major problem after curative resection of hepatocellular carcinoma (HCC). The current study evaluated the effects of adjuvant iodine-125 (^125^I) brachytherapy on postoperative recurrence of HCC.

**Methodology/Principal Findings:**

From July 2000 to June 2004, 68 HCC patients undergoing curative hepatectomy were randomly assigned into a ^125^I adjuvant brachytherapy group (n = 34) and a group of best care (n = 34). Patients in the ^125^I adjuvant brachytherapy group received ^125^I seed implantation on the raw surface of resection. Patients in the best care control group received identical treatments except for the ^125^I seed implantation. Time to recurrence (TTR) and 1-, 3- and 5-year overall survival (OS) were compared between the two groups. The follow-up ended in January 2010, and lasted for 7.7–106.4 months with a median of 47.6 months. TTR was significantly longer in the ^125^I group (mean of 60.0 months vs. 36.7 months in the control). The 1-, 3- and 5-year recurrence-free rates of the ^125^I group were 94.12%, 76.42%, and 73.65% vs. 88.24%, 50.00%, and 29.41% compared with the control group, respectively. The 1-, 3- and 5-year OS rates of the ^125^I group were 94.12%, 73.53%, and 55.88% vs. 88.24%, 52.94%, and 29.41% compared with the control group, respectively. The ^125^I brachytherapy decreased the risk of recurrence (HR = 0.310) and the risk of death (HR = 0.364). Most frequent adverse events in the ^125^I group included nausea, vomiting, arrhythmia, decreased white blood cell and/or platelet counts, and were generally mild and manageable.

**Conclusions/Significance:**

Adjuvant ^125^I brachytherapy significantly prolonged TTR and increased the OS rate after curative resection of HCC.

**Trial Registration:**

Australian New Zealand Clinical Trials Registry ACTRN12610000081011.

## Introduction

Partial hepatectomy is a potentially curative treatment for patients with hepatocellular carcinoma (HCC) [1]. However, the outcome after the surgery remains disappointing, mainly due to recurrence, which occurs in 40–80% of the patients within 5 years [2], [3]. Prevention of postoperative recurrence is pivotal in the improvement of surgical prognosis. Certain forms of adjuvant therapies, including transarterial lipiodol chemoembolization [4], [5], α-interferon [6], adoptive immunotherapy [7] and oral acyclic retinoid acid [8], have been reported as attempts to decrease the recurrence rate, but debates still remain.

Brachytherapy with radioactive seed implantation for the treatment of malignant tumors has been used for many years [9]. Radioactive seeds (e.g., cobalt-60 and radium-226) used in the early stage are nuclides that emit high-energy gamma rays. The high-energy irradiation prevented these agents from widespread use. Irradiation using low-energy chemicals (e.g., iodine-125 and palladium-103) has gained popularity in the past three decades [10]. Brachytherapy with interstitial implantation of radioactive seeds could achieve a high dose within the target area but the irradiation attenuates quickly over distance. In addition, brachytherapy is not affected by the body position and respiratory movements, thereby minimizing the possibility of geographic miss. The treatment has been recently used for a variety of cancers, including pancreatic cancer, pulmonary carcinoma, oral and maxillofacial tumors, and head and neck malignant neoplasms [11]–[16]. Therapeutic efficacy has been reported to be promising, particularly for prostate cancer [17], [18]. Treatment of liver metastasis from colorectal cancer with ^125^I implant has also been reported [19], [20]. To the best of our knowledge, no report of ^125^I implant as adjuvant treatment for HCC after surgery has been published in the English literature, although a preliminary study reported its use for inoperative HCC [21].

We conducted a randomized phase 2/3 clinical trial to examine whether adjuvant ^125^I brachytherapy could reduce tumor recurrence rate and increase overall survival (OS) rate in HCC patients after curative resection. Adverse reaction of this treatment was also examined.

## Methods

The protocol for this trial and supporting CONSORT checklist are available as supporting information; see Checklist S1 and Protocol S1.

### Participants

This is a single-center, open-label, randomized trial at a teaching hospital affiliated to a medical university. All patients were between 18 and 70 years of age, and had a chest x-ray, abdominal ultrasound and contrast computed tomography (CT) or magnetic resonance imaging (MRI) of the abdomen prior to the enrollment. The laboratory blood tests included hepatitis B and C virus antigen/antibodies, serum alpha-fetoprotein (AFP), carcinoembryonic antigen (CEA), carbohydrate antigen 19-9 (CA19-9), serum albumin (Alb), serum total bilirubin (Tbil), alanine aminotransferase (ALT) and prothrombin time (PT). The preoperative diagnosis of HCC was made by at least two radiological images showing characteristic features of HCC, or one radiological image showing characteristic features of HCC plus serum AFP at >400 ng/ml. Reserve liver function was estimated using Child-Pugh classification. HCC staging was determined according to the BCLC and 6^th^ TNM staging systems. Performance status was assessed with Karnofsky performance score (KPS).

The eligibility criteria included: (1) HCC patients who underwent curative hepatectomy; (2) KPS score >70; (3) Child-Pugh class A; (4) adequate bone marrow (white blood cell (WBC) count ≥4.0×10^9^/L, platelet (PTL) count ≥50×10^9^/L) and renal function (serum creatinine <1.5 mg/dL); (5) normal major organ (heart and lung) function; and (6) no previous anticancer treatment prior to the surgery. Curative hepatectomy for HCC was defined as: (1) complete tumor resection confirmed by intraoperative ultrasound and the clear resection margin verified by histological examination; (2) no gross tumor thrombus in the portal vein (main trunk or two major branches), hepatic veins or bile duct; (3) the number of tumor nodules ≤3; and (4) no extrahepatic metastasis. The patients were excluded if they had active thyroid disease, serious concurrent medical illnesses, histologically proved non-HCC tumors or they were pregnant or breastfeeding. The last follow-up was conducted on January 31^st^, 2010.

### Ethics Statement

The study was approved by the Institutional Ethics Committee of the Second Provincial People’s Hospital of Guangdong Province. The protocol was explained to eligible patients, and informed consent was obtained from all subjects before surgery. All participants were voluntary to enter the study and gave informed consent in writing.

### Hepatectomy

The surgical procedure was determined according to tumor size, anatomic location, reserve liver function and the estimated remnant liver volume/function. Standard operation included hemihepatectomy, lobectomy, segmental hepatectomy and wedge resection. Liver resection was carried out using a clamp-crushing method, and at least 1 cm surgical margin was retained. Intraoperative ultrasound was routinely employed. No operative death (death within the surgery or from complication within 30 days after the resection) occurred.

### Randomization

Randomization was performed in the operating room immediately after the curative resection. Eligible patients, who gave their consent to participate in the study, were randomly allocated into the adjuvant ^125^I brachytherapy group and the control group at 1∶1 ratio. The randomization procedure was done by computer-generated random numbers between 0 and 1 without stratification. Patients with odd number at the first decimal point were assigned to the ^125^I adjuvant brachytherapy group. Patients with even values (including zero) at the first decimal point were assigned to the control group. The allocation sequence was generated by Yong Xia. The participants were enrolled by Kaiyun Chen and Hanning Wang. Group assignment was carried out by Fanglian Xiao.

### Implantation of ^125^I Seeds

In the adjuvant ^125^I brachytherapy group, the wound was carefully stanched, the wound surface area was measured, and the data were recorded into the computer to determine the quantity and dose of seed implantation. The ^125^I seeds (0.8 mm in diameter and 4.5 mm in length) were enclosed in a NiTinol capsule (China Institute of Atomic Energy, Beijing). These seeds produce 27.4–31.5 keV X-ray and 35.5 keV γ ray, with a half-life of 59.6 days. The radioactivity per seed ranged from 0.5 to 0.6 millicuries (mCi). The megatemperature-sterilized seeds were implanted into the non-tumorous liver tissue adjacent to the cut surface with 1-cm intervals. The wound surface was covered by biological fibrin glue, followed by gelatin sponge or hemostatic gauze after saturation and fixation to prevent displacement of the ^125^I seeds. A median of 25 ^125^I seeds per patient (range: 18–34 seeds) were implanted, with a median activity of 0.5 mCi for a median total implanted activity of 12.5 mCi (range: 9.0–20.4 mCi). The implant volume ranged from 3–27 cm^3^ (median, 9 cm^3^). The estimate of volume in surface implants was performed by arbitrarily assuming a 1-cm thickness. The procedure in the control group was identical except that no ^125^I seed was implanted. Postoperative treatment was identical in the two groups.

### Follow-up and Outcome Measures

The follow-up included serum AFP assay, liver function test, abdominal ultrasound and chest x-ray every 2 months during the first 2 years after the surgery, and every 3 months afterwards. CT or MRI examination was performed every 3 months. If recurrence was suspected, hepatic angiography followed by Lipiodol computed tomography was performed. Recurrence was defined as lesions with typical findings of HCC on two or more imaging methods. The treatment for postoperative recurrence was based on the location, size and number of the recurrent tumors, as well as liver function. The last follow-up was conducted on January 31^st^, 2010.

The primary endpoint was the time to recurrence (TTR). The secondary endpoint was the OS. TTR was measured from the date of resection to the date when the diagnosis of recurrent tumor was established. Patients who died without recurrence were censored at their date of death. OS was calculated from the date of resection to the time of death or the follow-up. The hazard ratio (HR) for recurrence and death after the adjuvant treatment, as well as the adverse events (as measured using National Cancer Institute’s Common Toxicity Criteria) related to the adjuvant brachytherapy were also presented.

### Sample Size and Statistical Analysis

The median recurrence-free time or the median TTR after hepatectomy for HCC has been reported to be 13–24 months [22]–[25]. The study was designed to detect an increase in median TTR from 18 months in the control group to 36 months in the treatment group. The null hypothesis is: HR = 1.0. The alternative hypothesis is: HR = 0.5. The alpha error was set at 0.05, and the power was 0.80. The follow-up was planned for more than 5 years to cover potential variation from this estimate. The length of the accrual was expected to be at least 1 year. Therefore, in order to detect the difference, 34 patients were needed in each group.

All analyses were performed on an intention-to-treat (ITT) basis in a specialty hospital in the area of hepatobiliary diseases. Continuous data of normal distribution are presented as mean ± standard deviation (S.D.), and analyzed using Student’s t-test. Data of skew distribution are presented as median (range), and analyzed using the Mann-Whitney *U* test. Categorical data are presented as number (percentage), and analyzed using Chi-squared test. OS and TTR were estimated using Kaplan-Meier method and compared using a log-rank test. The effects of adjuvant ^125^I brachytherapy on recurrence and OS were estimated using a Cox proportional hazards model. A univariate analysis was performed to identify factors that could affect the outcomes. P-values were not adjusted for multiple testing. Factors with *p*<0.2 were entered into a multivariate analysis for identification of independent prognostic factors. For TNM stage, the statistical analysis was carried out using two dummy variables, with reference to TNM stage I. Data were analyzed by Statistical Package for the Social Sciences software (SPSS Inc., Chicago, IL, USA), and *p*<0.05 was considered statistically significant.

## Results

### Participant Flow and Baseline Characteristics

**Figure 1:**
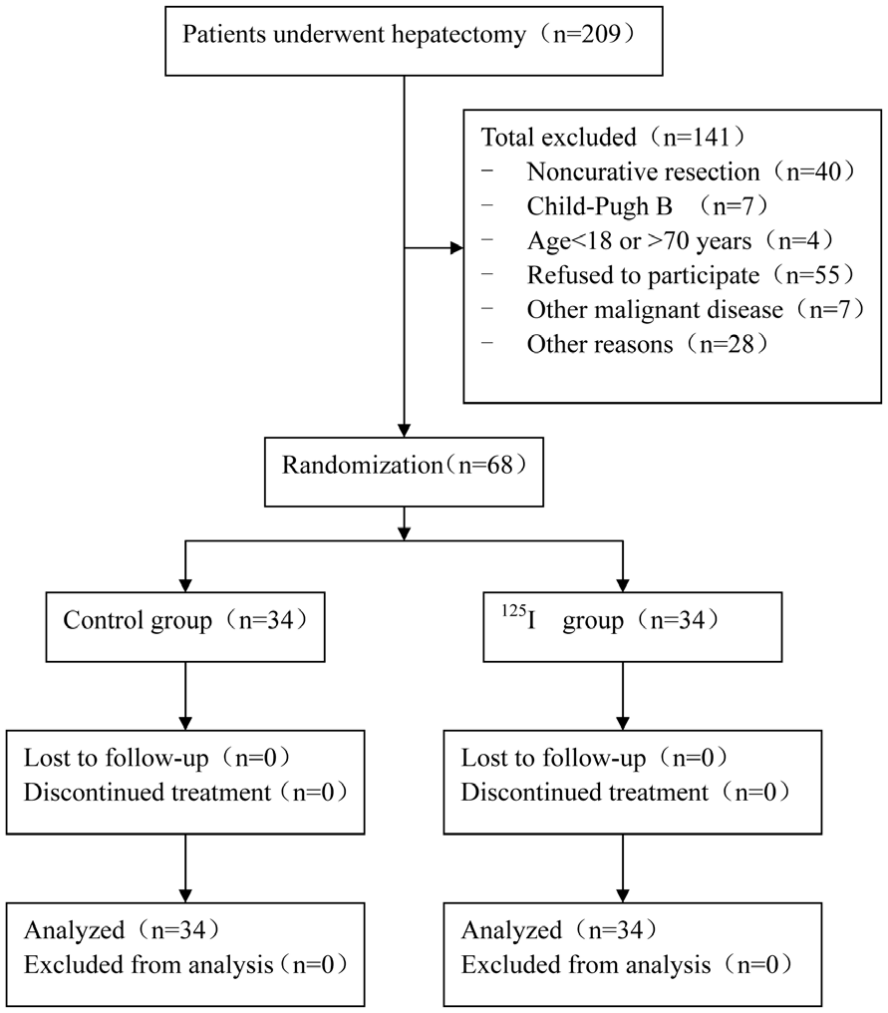
A flow chart of the trial.

A total of 209 consecutive patients receiving curative liver resection for HCC from July 2000 to June 2004 were screened. We excluded 141 cases for a variety of reasons (Figure 1). The remaining 68 patients were equally and randomly assigned into the two groups. Table 1 shows the clinical and pathological characteristics of these participants. The median follow-up duration was 47.6 months (range: 7.7–106.4 months), with no patient lost to the follow-up.

**Table 1:** Patient characteristics.

Factors	Control group	^125^I group	*p* value
	(n = 34)	(n = 34)	
Male/Female	24/10	25/9	0.787
HBs-Ag Positive (%)	31 (91.2%)	26 (76.5%)	0.100
HBe-Ag Positive (%)	12 (35.3%)	14 (41.2%)	0.618
HCV-Ab Positive (%)	5 (14.7%)	6 (17.6%)	0.742
Liver cirrhosis (%)	20 (58.8%)	18 (52.9%)	0.625
Age (years) mean (SD)	48.91 (7.30)	50.79 (6.79)	0.275
Total bilirubin (µmol/L) mean (SD)	13.97 (3.44)	14.07 (3.10)	0.903
Albumin (g/L) mean (SD)	42.21 (3.91)	41.29 (3.16)	0.294
ALT (U/L) mean (SD)	24.65 (9.32)	26.18 (9.18)	0.498
AFP (ug/L) mean (SD)	579.26 (298.46)	611.97 (265.94)	0.635
Prothrombin time (second) mean (SD)	14.30 (.98)	14.17 (1.06)	0.595
Child-Pugh Class A/B	34/0	34/0	1.000
Mean tumor size (cm) mean (SD)	5.65 (2.52)	6.24 (2.55)	0.342
Number of tumors			0.690
single (%)	31 (91.2%)	30 (88.2%)	
multiple (%)	3 (8.8%)	4 (11.8%)	
Tumor encapsulation			0.120
absent (%)	8 (23.5%)	14 (41.2%)	
present (%)	26 (76.5%)	20 (58.8%)	
BCLC stage			0.690
0-A	31 (91.2%)	30 (88.2%)	
B	3 (8.8%)	4 (11.8%)	
TNM stage			0.873
I (%)	20 (58.8%)	18 (52.9%)	
II (%)	11 (32.4%)	13 (38.2%)	
III a (%)	3 (8.8%)	3 (8.8%)	
Microscopic vascular invasion			0.465
no (%)	20 (58.8%)	17 (50.0%)	
yes (%)	14 (41.2%)	17 (50.0%)	
Edmondson-Steiner’s grade			0.988
I (%)	10 (29.4%)	9 (26.5%)	
II (%)	13 (38.2%)	13 (38.2%)	
III (%)	6 (17.6%)	7 (20.6%)	
IV (%)	5 (14.7%)	5 (14.7%)	
Surgical procedure			0.702
wedge resection (%)	0	1 (2.9%)	
Subsegmentectomy (%)	1 (2.9%)	1 (2.9%)	
Segmentectomy (%)	25 (73.5%)	22 (64.7%)	
Lobectomy (%)	8 (23.5%)	10 (29.4%)	
Surgical margin			1.000
<2 cm (%)	5 (14.7%)	5 (14.7%)	
≥2 cm (%)	29 (85.3%)	29 (85.3%)	
No. transfusion (%)	5 (14.7%)	9 (26.5%)	0.230
Blood loss (ml) median (range)	400 (200–800)	350 (100–700)	0.478
Blood transfusion (ml) median (range)	0 (0–400)	0 (0–400)	0.284

### Adverse Events

Adverse events of all subjects were generally mild (Table 2). Most frequent adverse events in the adjuvant ^125^I brachytherapy group included: nausea in four patients (including two with vomiting), arrhythmia in six patients (four with sinus tachycardia, one with frequently premature atrial contraction and one with premature ventricular contraction), and decreased WBC count (<3×10^9^/L) and/or decreased PTL count (<40×10^9^/L) in three patients. Nausea dissipated after symptomatic treatment. The patients with decreased WBC and/or PTL count were treated with 20 mg 2-(a-phenylethylacetate)-4-carboxylthiazolidine and 50 mg batylalcohol (three times a day). The WBC and/or PTL count restored to the normal range within one week. Tachyarrhythmia usually occurred within 1–5 postoperative days in patients, and dissipated after about 1 week of symptomatic treatment. No hepatic failure was observed prior to tumor recurrence in any patient.

**Table 2:** Adverse events.

Adverse events	Grade I and II		Grade III and IV			
	Control group (n = 34)	^125^I group (n = 34)	Control group (n = 34)		^125^I group (n = 34)	
Fever	2	2	0	0		
Nausea	2	3	1	1		
Vomiting	3	2	0	0		
Diarrhea	3	0	0	0		
Hair loss	1	1	0	0		
Sinus tachycardia	1	3	0	1		
Premature atrial contraction	1	0	0	1		
Premature ventricular contraction	0	1	0	0		
Decreased WBC and/or platelets	3	3	1	0		
Dermatitis	1	0	0	0		

### Tumor Recurrence

**Figure 2:**
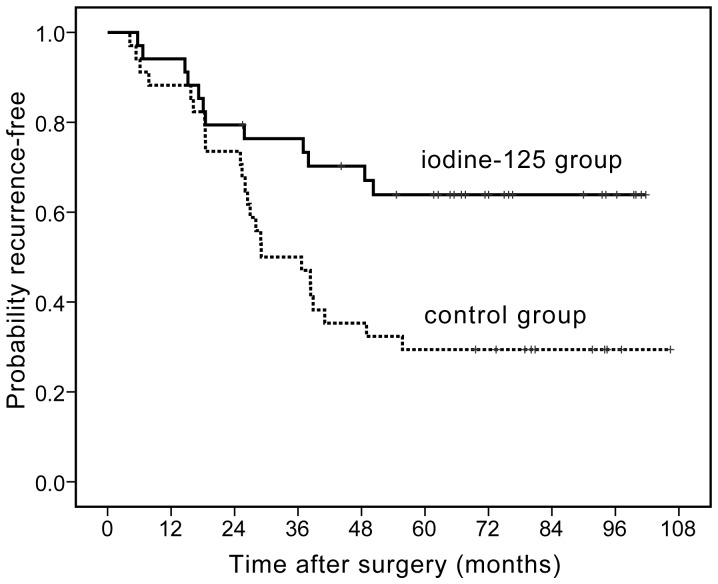
Time to recurrence curves. Time to recurrence in patients in the adjuvant ^125^I brachytherapy vs. in the control group, log-rank, *X* ^2^ = 7.04, *p = *0.008.

At recurrence, the tumor was within the liver, with no extrahepatic recurrence in all cases. TTR in the adjuvant ^125^I brachytherapy group had a mean of 60.0 months (vs. 36.7 months in the control group). The rate of postoperative recurrence was 35.29% (12/34 patients) in the adjuvant ^125^I brachytherapy group as opposed to 70.59% (24/34 patients) in the control group (Figure 2). The 1-, 3- and 5-year recurrence-free rates of the ^125^I group were 94.12%, 76.42% and 73.65% vs. 88.24%, 50.00% and 29.41% of that in the control group (log-rank test, *p* = 0.008), respectively. There were two recurrence-free deaths in the adjuvant ^125^I brachytherapy group, which were also included and censored on the date of death in the Kaplan-Meier plot for the recurrence-free rate. The comparison of recurrence-free rate between the adjuvant ^125^I brachytherapy group and control group is presented in Figure 2.

Upon univariate analysis, pTNM stage, liver cirrhosis and adjuvant ^125^I brachytherapy were prognostic factors that affected the recurrence. Multivariate analysis indicated that pTNM stage and adjuvant ^125^I brachytherapy are independent prognostic factors. The risk of recurrence in the adjuvant ^125^I brachytherapy group was lower than that in control group **(**HR = 0.310, [95% confidence intervals (CI), 0.145–0.662], Cox proportional hazards regression model, *p* = 0.002) (Table 3 and Table 4).

**Table 3:** Univariate analyses of the recurrence- and OS-related factors.

Factors	Number of cases with recurrence (%)	*p* value: log-ranktest for recurrence	Number ofdeath (%)	*p* value: log-ranktest for OS
Gender				
male (n = 49)	26 (53.1)	0.912	27 (55.1)	0.780
female (n = 19)	10 (52.6)		12 (63.2)	
Age				
<55 years (n = 49)	26 (53.1)	0.952	27 (55.1)	0.643
≥55 years (n = 19)	10 (52.6)		12 (63.2)	
Intervention				
^ 125^I (n = 34)	12 (35.3)	0.008	15 (44.1)	0.026
control (n = 34)	24 (70.6)		24 (70.6)	
HBs-Ag				
negative (n = 11)	3 (27.3)	0.115	4 (36.4)	0.163
positive (n = 57)	33 (57.9)		35 (61.4)	
HBe-Ag				
negative (n = 42)	22 (52.4)	0.894	24 (57.1)	0.870
positive (n = 26)	14 (53.8)		15 (57.7)	
HCV-Ab				
negative (n = 57)	29 (50.9)	0.420	32 (56.1)	0.557
positive (n = 11)	7 (63.6)		7 (63.6)	
Liver cirrhosis				
no (n = 30)	11 (36.7)	0.033	13 (43.3)	0.049
yes (n = 38)	25 (65.8)		26 (68.4)	
Total bilirubin level				
≤17.1 µmol/L (n = 55)	28 (50.9)	0.291	30 (54.5)	0.224
>17.1 µmol/L (n = 13)	8 (61.5)		9 (69.2)	
Prothrombin time				
≤15s (n = 55)	29 (52.7)	0.927	30 (54.5)	0.224
>15s (n = 13)	7 (53.8)		9 (69.2)	
AFP				
<400 ng/mL (n = 21)	8 (38.1)	0.250	10 (47.6)	0.456
≥400 ng/mL (n = 47)	28 (59.6)		29 (61.7)	
Tumor size				
≤5.0 cm (n = 32)	17 (53.1)	0.717	17 (53.1)	0.402
>5.0 cm (n = 36)	19 (52.8)		22 (61.1)	
Tumor number				
single (n = 61)	32 (52.5)	0.230	34 (55.7)	0.103
multiple (n = 7)	4 (57.1)		5 (71.4)	
Tumor encapsulation				
absent (n = 22)	8 (36.4)	0.133	10 (45.5)	0.265
present (n = 46)	28 (60.9)		29 (63.0)	
BCLC stage				
0-A (n = 61)	32 (52.5)	0.230	34 (55.7)	0.103
B (n = 7)	4 (57.1)		5 (71.4)	
TNM stage		0.050		0.013
I (n = 38)	17 (44.7)		18 (47.4)	
II (n = 24)	15 (62.5)		16 (66.7)	
III a (n = 6)	4 (66.7)		5 (83.3)	
Microscopic vascular invasion				
no (n = 37)	18 (48.6)	0.240	19 (51.4)	0.139
yes (n = 31)	18 (58.1)		20 (64.5)	
Edmondson-Steiner’s grade				
I (n = 19)	9 (47.4)	0.623	10 (52.6)	0.427
II (n = 26)	14 (53.8)		14 (53.8)	
III (n = 13)	8 (61.5)		9 (69.2)	
IV (n = 10)	5 (50.0)		6 (60.0)	
Hepatectomy procedure				
minor resection (n = 50)	26 (52.0)	0.545	27 (54.0)	0.259
major resection (n = 18)	10 (55.6)		12 (66.7)	
Surgical margin				
<2 cm (n = 10)	4 (40.0)	0.295	5 (50.0)	0.411
≥2 cm (n = 58)	32 (55.2)		34 (58.6)	
Blood transfusion				
no (n = 54)	31 (57.4)	0.300	32 (59.3)	0.623
yes (n = 14)	5 (35.8)		7 (50.0)	

**Table 4:** Multivariate analyses of the recurrence- and OS-related factors.

Independent factors	B	SE	Wald	Significance	HR	95.0% CI for HR	
						Lower	Upper
Recurrence							
^ 125^I brachytherapy	−1.171	0.387	9.167	0.002	0.310^a^	0.145	0.662
pTNM stage			10.201	0.006			
pTNM II vs I*	0.563	0.355			1.755^b^	0.875	3.523
pTNM IIIa vs I*	1.911	0.614			6.758^c^	2.209	22.515
Overall survival							
^ 125^I brachytherapy	−1.012	0.364	7.742	0.005	0.364^d^	0.178	0.741
pTNM stage			13.010	0.001			
pTNM II vs I*	0.609	0.345			1.839^e^	0.936	3.613
pTNM IIIa vs I*	1.984	0.561			7.274^f^	2.421	21.857

a, d the HR is the ratio of brachytherapy to control.

b, c, e, f the HR is the ratio of higher to lower stage.

* TNM was a categorical variable that was assessed using dummy variables with stage I as the reference.

### Overall Survival

**Figure 3:**
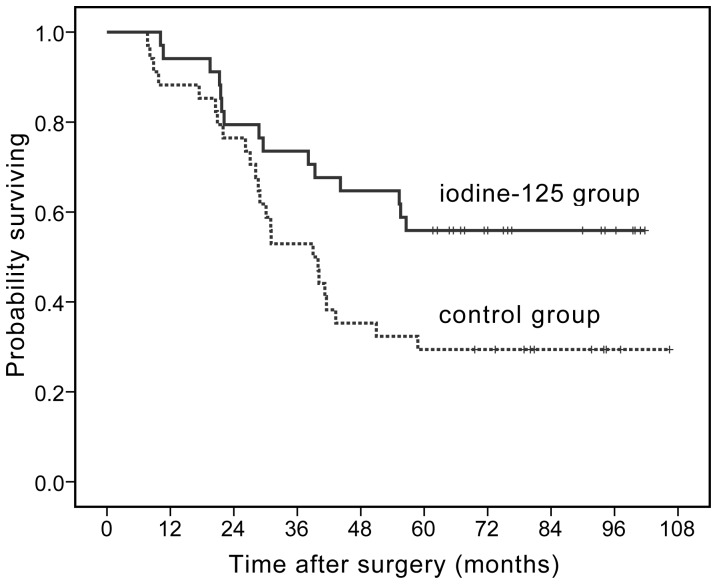
Overall survival curves. OS in patients in the adjuvant ^125^I brachytherapy vs. in the control group, log-rank, *X* ^2^ = 4.97, *p* = 0.026.

Fifteen and 24 patients (44.11% and 70.59%) died in the adjuvant ^125^I brachytherapy and the control groups, respectively. Major causes of death included hepatic and renal failure as well as cachexia resulting from tumor recurrence, with the exception of cerebrovascular accidents and coronary heart disease in two cases in the adjuvant ^125^I brachytherapy group. The mean OS was 63.6 months and 38.9 months in the adjuvant ^125^I brachytherapy group and the control group, respectively. The 1-, 3- and 5-year OS rates of the adjuvant ^125^I brachytherapy group and control group were 94.12%, 73.53% and 55.88% vs. 88.24%, 52.94% and 29.41% (Kaplan-Meier, log-rank test, *p* = 0.026, Figure 3), respectively. The OS rate of the adjuvant ^125^I brachytherapy group was significantly higher than that of the control group.

Univariate analysis showed that pTNM stage, liver cirrhosis and adjuvant^ 125^I brachytherapy significantly affected the OS. A multivariate analysis showed that pTNM stage and adjuvant ^125^I brachytherapy are independent prognostic factors that affected the OS. Tables 3 and 4 show that HR for death of the adjuvant ^125^I brachytherapy group was 0.364 relative to that in the control group (95% CI, 0.178–0.741, Cox proportional hazards regression model, *p* = 0.005).

## Discussion

Results from the current study indicated that ^125^I brachytherapy could increase TTR and OS in HCC patients receiving curative resection. The risk of tumor recurrence was also significantly decreased by the ^125^I brachytherapy (HR = 0.310). The risk of death in the adjuvant ^125^I brachytherapy group was significantly lower than that in the control group (HR = 0.364). Considering the relatively mild adverse events, ^125^I brachytherapy is a useful and safe adjuvant therapy after curative resection of HCC in our opinion.

Previous attempts to improve the long-term outcome with adjuvant therapy after curative resection for HCC have been generally disappointing [26]. Systemic and regional chemotherapies failed to show significant survival benefit in randomized trials [4], [27]. Other adjuvant modalities, including subcutaneous α-interferon injection, oral acyclic retinoic acid, intra-arterial lipiodol-iodine-131 and adoptive immunotherapy, have shown promising results [6]–[8], [24], [25], but remain controversial for HCC [28]. ^125^I seeds have been used for a variety of tumors, including prostatic carcinoma, pancreatic carcinoma, lung cancer, oral and maxillofacial malignant tumors, and malignant tumors of the head and neck [12], [16], [17], [29]. Some studies reported prolonged survival after implanting ^125^I seed into the tumor tissue or tumor bed in patients with metastatic liver cancer [19]. For advanced unresectable HCC, CT-guided ^125^I seed intrahepatic implantation may achieve higher rate of complete and partial remission [21]. TACE in combination with portal vein stent and ^125^I implantation may be safe and effective for HCC with tumor thrombus in the main portal vein [30], [31]. To the best of our knowledge, no study of adjuvant ^125^I brachytherapy for resectable HCC has been published in the English literature.

Implantation of permanent radioactive ^125^I seeds offers the advantage of intraoperative placement under direct vision. The seeds could produce radiation to the remaining cancer cells from a very short distance. The irradiation decreases sharply from the center to the periphery, with only 1% dosage at 5 cm from the source (relative to 1 cm from the source) [32], thus limiting the exposure to other vital organs previous studies have also indicated that radiosensitivity is cell cycle dependent, and cells in the G2/M phase are more radioresponsive [33]. Continuous irradiation at low dose, such as obtained with ^125^I seeds, enhances the radiosensitivity by inducing the accumulation of cells in a more radiosensitive cell cycle phase (G2/M) and results in more tumor cell destruction [34]. A previous study from this laboratory showed ^125^I implantation stimulates the anti-tumor immune response in HCC patients by increasing CD3^+^ and CD4^+^ immunocytes and promoting Th2/Th1 deviation [35]. Together, these findings suggested that ^125^I brachytherapy could target tumor cells more effectively and minimize damage to healthy tissues, including the remaining liver.

Adverse events of adjuvant ^125^I brachytherapy are generally related to exposure to radioactive material, but are generally mild and manageable. Similar to the previous studies [19], [20], most frequent adverse events in the ^125^I group included nausea/vomiting, leukocytopenia and thrombocytopenia. Cardiac arrhythmia occurred in a few patients but spontaneously dissipated with no treatment. The result suggested that cardiac and pulmonary function need to be evaluated vigorously. However, this study is limited in several aspects. First, the sample size is relatively small. Second, we did not attempt stratified analysis that could be obvious to the readers (such as based on tumor size). Thirdly, the multiple testing was not adjusted. Multiple testing without adjustment increases the probability of finding a statistically “significant” change by chance alone.

In conclusion, ^125^I implant into the cut surface of remnant liver is an effective adjuvant modality for HCC patients after the radical hepatectomy. Our results demonstrated that ^125^I brachytherapy is a safe and could delay postoperative tumor recurrence. Multi-center, randomized controlled trials of larger scale are essential to verify these findings.

## Supporting Information

Checklist S1 **The checklist that items pertain to the content of the Title, Abstract, Introduction, Methods, Results, Discussion, and Other information.** Details of these items, as found in the CONSORT 2010 Explanation and Elaboration document, can be browsed using the menu on the left.
(DOC)

Protocol S1 **A copy of the trial protocol as approved by the ethics committee.**
(PDF)
