# Supplementary material for: Adjuvant Iodine-125 Brachytherapy for Hepatocellular Carcinoma after Complete Hepatectomy: A Randomized Controlled Trial
Source: PLoS One. 2013 Feb 28;8(2):e57397. doi: 10.1371/journal.pone.0057397 (PMC3585398; doi:10.1371/journal.pone.0057397)
Supplement: Protocol S1 — A copy of the trial protocol as approved by the ethics committee. (PDF) [file pone.0057397.s002.pdf]

Implantation of radioactive seeds Iodine-125 for anti-recurrence after curative resection of hepatocellular carcinoma: A prospective randomized control trial

Chief person in-charge of the study: XIANG GuoAn, SHEN Feng

Affiliations: Guangdong Second People's Hospital
Shanghai Eastern Hepatobiliary Hospital

Telephone: 86-13138631120

E-mail: guoanxiang@yahoo.cn

1. Background

The mortality and morbidity of Hepatocellular carcinoma (HCC) ranks first and third in refractory carcinoma, respectively. China is a high incidence country of HCC. The standard mortality rates of HCC of male adults and female adults were 14.52/100,000 and 5.61/100,000, respectively. That of HCC in such high incidence areas as QiDong, TongAn, ShunDeand etc. was over 30/100,000. Therefore, prevention and cure of HCC are severe in China.

With great accomplishment having been achieved, increase of early diagnosis of HCC and emergence of new treatment and technology for HCC, combined treatments for HCC based on surgery has been applied in clinical. However, difficulty in early diagnosis, low cure rates of the first operation, high recurrence rates and metastasis rates after a curative operation still exist without improvement. Especially recurrence of HCC after the curative operation is still an essential problem hindering improvements of surgeries for HCC.

1.1 Treatment for HCC after a surgery

Those include prevention and treatment for recurrence, in which the anti-recurrence treatment (adjuvant treatment after a surgery) including TACE, immunological treatment and immunoradiation treatment have been studied in recent years. Nevertheless, most of their effects have not been completely proved so far.

Effects of TACE have been proved in late stage of HCC, but without proof as an adjuvant method, mainly owing to inconsistence of conclusions of retrospective and prospective studies. In prospective studies, researchers found that TACE produced no effects with regard to anti-recurrence after the surgery even did harm. Lai etc. combined iodized oil, cis-platinum and epirubicin to treat HCC. Compared with the control group, there was no difference in the overall 3-year survival rates (66% vs. 65%) of the two groups. The 3-year tumor-free survival rate of treated patients was (18%) lower than that of patients in the control group (48%). However, most results of retrospective studies showed that TACE was effective in prevention of recurrences as an adjuvant method after the surgery. According to a retrospective study of 987 patients treated with TACE as an adjuvant method in Eastern Hepatobiliary Hospital, TACE prevented recurrence in some of patients with high recurrence. The recurrence rates of the study group and the control group were 22.2% and 61.1%, respectively.

Immunological treatment shows its potential value, but exact efficacy needs to be further tested and proved and its standards have not been established. Compared with other tumor, HCC has a clear relationship with infection of HBV, so theoretically, immunological treatment (thymic peptide and interferon) could improve immunity against tumor and hepatic virus, thus reducing recurrence rates. It was reported that tumor-free and overall survival rates of some of subtypes of HCC could be increased by interferon. Activity of hepatitis affected survival of the patients and antiviral treatment extended tumor-free survival after surgeries. However, those results have not been proved by large sample clinical trials.

Studies of radionuclide carriers with ^{90}Y, ^{131}I and etc. in TACE initially proved their prevention of recurrence. Associated prospective randomized control trials with large samples are under progress.

1.2 Study of antitumor effect of ^{125}I

Implantation of radionuclides within tissues for radiotherapy against carcinoma has been hundreds years. Radionuclides in early time were ^{60}Co, ^{226}Ra and etc. with high energy γ-ray, a serious health hazard to patients and medical personnel who were hard to be protected, resulting in

restrictions of applications in clinical. In recent 20 years, such new low energy radionuclides as ^{125}I, ^{103}Pd and etc. have widened indications of radiotherapy. Implantation of radionuclides within tissues could produce local radiation, as a result, target areas receive maximum doses of rays and doses in surrounding normal tissues dramatically decrease, which reduces side effects. In addition, target areas don’t change with respiratory and body positions, so loss of volumes of tumor receiving radiotherapy significantly decrease. Recently, ^{125}I has been applied in treatment of some carcinoma, such as pancreatic cancer, lung cancer, oral and maxillofacial cancer, head and neck cancer and etc. Radiotherapy is particularly effective in prostate cancer. With respect to liver foci there are some studies on implantation of^{125}I to treat liver metastasis of colon cancer but no studies on ^{125}I for anti-recurrence after surgeries for HCC.

In order to assess the efficacy of implantation of^{125}I as an adjuvant method to treat HCC indicating surgeries before, a randomized control trial was run to see whether ^{125}I could prevent recurrence after curative surgery of HCC, improve prognosis and reveal its side effects at the same time.

2. Objectives

(1) To assess the efficacy and safety of prevention of recurrence of ^{125}I after surgeries for HCC.

(2) To develop a rational, effective and standardized adjuvant treatment protocol of prevention of recurrence with ^{125}I after surgeries of HCC.

3. Flow diagram

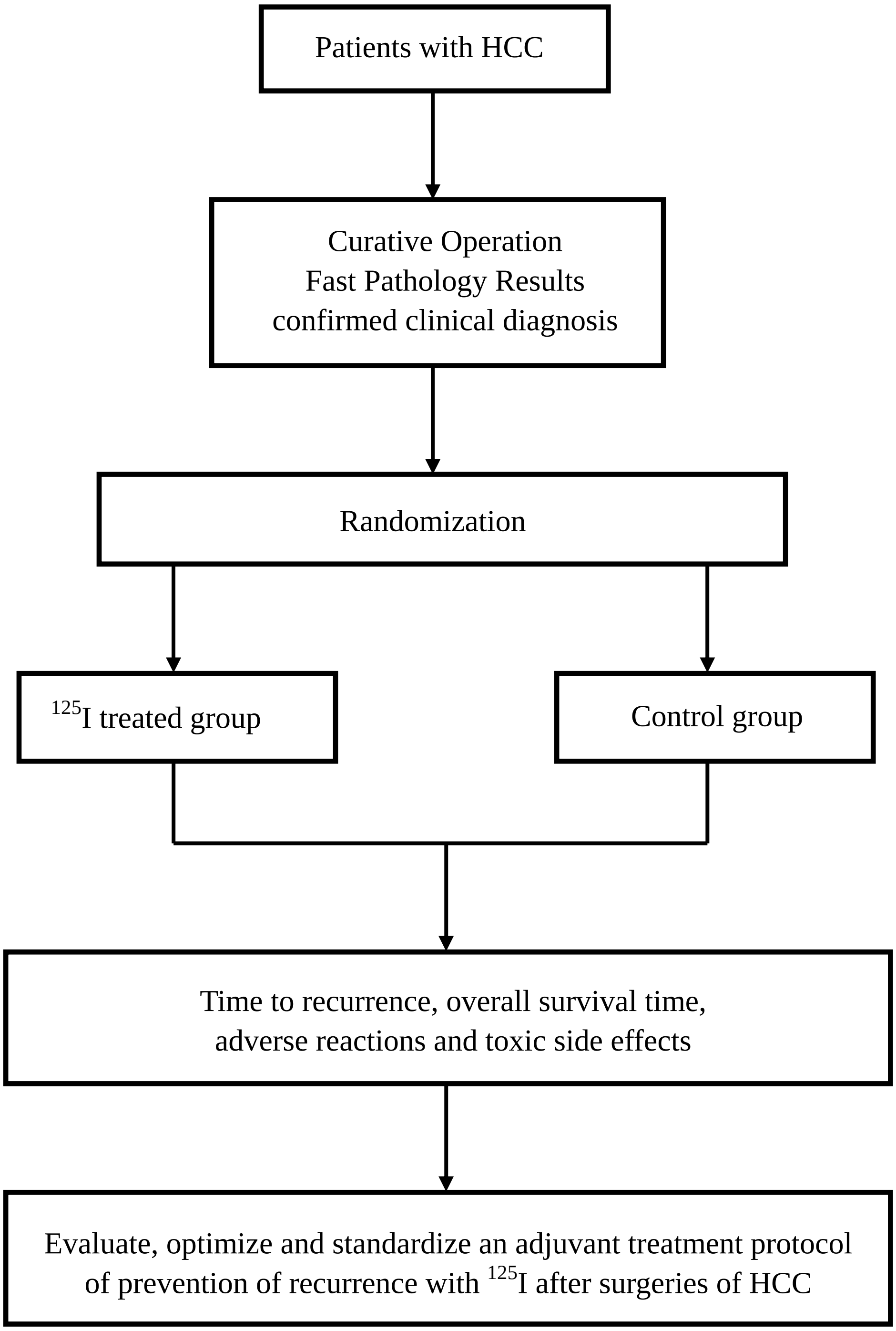

4. Introduction of the study

4.1 Study Design

It is a prospective randomized clinical control trial, assessing the efficacy and safety of prevention of recurrence of ^{125}I after surgeries for HCC, from which a rational, effective and standardized adjuvant treatment protocol of prevention of recurrence with ^{125}I after surgeries of HCC could be developed.

4.2 Population and Period

The total number of cases is 68.

It was reported that the median tumor-free survival time or median recurrence time was 13-24 months after a curative surgery for HCC. In this study, the median recurrence time of the control group is supposed to be 18 months. That of the group of patients with implantation of ^{125}I is expected to be 36 months (the hazard ratio of ^{125}I was 0.5). A probability value of <0.05 in one-sided test is considered statistically significant. Power of the test was 0.80. The number of cases in the study is 68.

These patients will be treated in our hospital from July 2000 to June 2004. The follow-up period was extended to 5 years after surgery.

4.3 Grouping

All of the patients will be grouped after receiving curative surgeries for HCC.

^{125}I treated group: The wound will carefully stanch after the tumor have been removed completely. The square meter of the wound will be measured and the data will be input into the computer to determine the number of radionuclides will implant and doses. Mick radionuclide applicator will be used to implant ^{125}I at 1cm interval in the normal liver tissues at the incisal margin (^{125}I was produced in Reactor Engineering Research of China Institute of Atomic Energy with 0.8mm× 4.5mm and a half-life of 59.6 days and enveloped with Nickel titanium alloy. It is sterilized under high temperature. It has X- ray with energy of 27.4-31.5Kev and γ -ray with energy of 35.5 Kev. The activity of per seed is 0.5-0.6mCi). Ultrasonography is utilized to see whether seeds are well-distributed. More seeds will be implanted so as to avoid cold spot areas if seeds are not well-distributed. Fibrin gel will be smeared on wounds and then sutured with absorbable gelatin sponge or hemostatic gauze to prevent ^{125}I from dropping off.

Control group: The rest treatment will be the same as that of the ^{125}I treated group except for implantation of ^{125}I.

Both of the groups received identical routine support treatment for the liver after surgery.

All of the patients will receive venous blood collection on one day before surgeries and day 7 after surgery. Aminotransferase and bilirubin were measured. AFP and MMP-9 will be measured via radioimmunoassay. Meanwhile, activity of nature killer cells and T cell subsets will be measured by flow cytometers (America COULTER). Curative hepatectomy of HCC must meet these following conditions: a. Tumor is completely removed. Explorations during surgery show no tumor remaining and fast pathology results revealed that there are no cancer cells in the incisal margins. b. There are no macroscopic cancer emboli in the main side branches and bile duct bifurcations. c. The number of the tumor is less than three. d. There is no extrahepatic metastasis.

4.4 Randomization

Patients included in the study will be allocated randomly. Random digits will be generated by computer. Custody of the random number table by hand.

4.5 Patients

4.5.1 Inclusion criteria

(1) Volunteer patients with good compliance and ranging from 18 to 70 years.

(2) Patients meeting these following conditions:

- Tumor has been removed completely via evaluations.
- The pathological diagnosis is HCC.
- The maximum diameter of a single tumor is less than or equal to 10cm and greater than or

equal to 2cm.

- The number of the tumor is less than three and the maximum diameter is less than or equal to 5cm regarding multiple nodule.
- No macroscopic vessel invasion.

(3) Other functions of organs are normal.

(4) KPS is over 70.

(5) Child-pugh stage A.

(6) The latest results of laboratory tests should meet these following standards:

- HgB≥9.0g/dl.
- The count of neutrophils is more than 1,500/mm.
- The count of platelets is more than 50,000/ul.
- The levels of ALT and AST are lower than or equal to twice normal ceiling values.
- The level of serum creatinine is less than or equal to 1.5 times normal ceiling values.

(7) The HCG test of women with fertility should be negative within 14 days before treatment.

(8) Patients who meet all the above conditions sign written informed consents.

4.5.2 Exclusion criteria

(1) Patients under 18 or over 70.

(2) Pregnant women or women in lactation.

(3) Poor physical conditions and liver functions (Child-pugh stage B and C, refractory ascites and brain diseases).

(4) Severe brain, lung, kidney and hematological diseases.

(5) Any unstable factors or factors affecting subjects' safety or compliance.

(6) Patients with known or doubtful allergic to iodine.

(7) Patients received other treatment for tumor before surgeries (such as liver transplantation, TACE, local ablation for HCC, chemotherapy, radiotherapy and etc.).

(8) Patients participating in other trials.

(9) Patients with heart diseases: CHF>NYHA stage 2, coronary atherosclerotic heart disease, arrhythmia or treated arrhythmia with medicine (digoxin or β-receptor blocker were allowed) and refractory hypertension.

(10) Severe active infections.

(11) Epilepsy treated with medicine.

(12) Patients who have received allograft transplantations.

(13) Patients with history of other tumor except that he or she have been cured 3 years before the study.

(14) Patients with history of bleeding or susceptibility to bleeding.

(15) Patients meanwhile receiving dialysis.

(16) Recurrent HCC.

5. Method

5.1 Radionuclides

5.1.1 ^{125}I

^{125}I is produced in Reactor Engineering Research of China Institute of Atomic Energy with 0.8mm ×4.5mm and a half-life of 59.6 days and enveloped with Nickel titanium alloy. It is sterilized under high temperature. It has X- ray with energy of 27.4-31.5Kev and γ-ray with energy of 35.5

Kev. The activity of per seed is 0.5-0.6mCi.

5.2 Treatment and doses

5.2.1 Hepatectomy

The extents of resection depended on volumes and positions of tumor, estimated rest liver volumes and stages. Standard approaches for surgery will be adopted, such as hemihepatectomy, hepalobectomy, hepatic segmentectomy and wedge resection of the liver, which are via finger pinching or jaws. During resections, the incisal margins should be over 1cm and ultrasonography was routinely utilized. The wounds will suture or covere with the free omentum. Double cannula drainage will be placed next to the wounds after the surgeries.

5.2.2 Interventions of two groups after removal of the tumor

^{125}I group: The wound will be carefully stanched after the tumor has been removed completely. The square meter of the wound will be measured and the data will be input into the computer to determine the number of radionuclides implanted and doses. Mick radionuclide applicator will be used to implant ^{125}I at 1cm interval in the normal liver tissues at the incisal margin (^{125}I was produced in Reactor Engineering Research of China Institute of Atomic Energy with 0.8mm× 4.5mm and a half-life of 59.6 days and enveloped with Nickel titanium alloy. It is sterilized under high temperature. It has X- ray with energy of 27.4-31.5Kev and γ -ray with energy of 35.5 Kev. The activity of per seed is 0.5-0.6mCi. The prescription dose was 160Gy). Ultrasonography is utilized to see whether seeds are well-distributed. More seeds will be implanted so as to avoid cold spot areas if seeds are not well-distributed. Fibrin gel will be smeared on wounds and then sutured with absorbable gelatin sponge or hemostatic gauze to prevent ^{125}I from dropping off. The rest treatment of the control group is the same as that of the ^{125}I treated group except for implantation of ^{125}I. Both of the groups will receive identical routine support treatment for the liver after surgeries.

6. Concomitant medication

The use of drugs or treatment during the study period

The use of concomitant drug and treatment during the study period according to the need is as follows:

- When the test drug induced adverse effects or the disease is in need of treatment, drugs can be given for relieving the symptoms.
- When the subjects vomite, which is caused by chemotherapy drugs, the antiemetic can be given.
- When the pain of subjects influenced sleep and rest, painkillers can be given.
- When the subjects have constipation, diarrhea, insomnia and other symptoms because of other reasons, drugs cant be given for relieving symptoms.
- When the subjects have anaphylactoid reactions, HAMA response and infusion reactions, dexamethasone can be given.
- Furosemide could be used.
- Routine liver protecting and immune improving drugs can be use.
- Antiviral drugs can be used for anti-viral treatment.
- Routine postoperative treatment, such as TACE, PEIT, and local ablation therapy can be carried out.

All the simultaneously used drugs should be detailed recorded in CRF, and described.

7. Management of test chemicals

7.1 Management of ^{125}I seeds

a) Radioactive particles must be used by experienced physicians in hospitals with relevant qualifications.

b) The seeds should be strictly in accordance with the indications and usage of the program recommending dosage range, which was not free to be changed.

7.2 Custody and distribution systems

7.2.1 The radioactive seeds should be kept by the person responsible for the custody in accordance with national drug regulations.

Registration statistical forms should be established with serious filling and permanent preservation.

If the product is found lost after being marked, it should be immediately traced and reported to the higher authorities.

Before the product is used for patients, the name of the seeds, patient's name and dosage should be strictly and carefully checked.

7.2.2 The procedure of preservation and distribution

Each patient of the group will obtain a random number, and then the number will be reported to the preservers of the center by the researchers. The preservers would judge whether to use seeds which will be distributed to the patients with the corresponding number.

8. Observed indicators and inspection time

8.1 Examinations before entering the groups

- Physical examination, vital signs and physical status score;
- Blood routine, liver and renal function;
- Hepatitis virus;
- ECG;
- Imaging studies: (B ultrasound must be carried out; enhanced abdominal CT or MRI was needed) In order to reduce the detection error, the method of examination of the tumor lesion hospitals and the equipments for use must be consistent;
- Serum tumor markers: AFP, CEA, and CA199.

8.2 Examinations during the study

- Physical examination, vital signs and behavioral state classification;
- Blood, urine routine;
- Liver and kidney function;
- Imaging studies;
- Tumor markers (such as: AFP, CEA, and CA199).

The above examinations could be carried out every 2 to 3 months in the treatment hospitals or local hospitals.

9. Follow-up

Patients must be arranged for close follow-up which included rechecks every 2-3 months or contact by phone after entering the treatment group.

Follow-up will be carried out according to the following stipulations with CRF filling:

Every 2to 3 months: blood routine, liver and kidney function, AFP, liver ultrasonography;
Every 3 to 4 months: enhanced abdominal CT or MRI.
Every six months: chest X-ray or chest CT;
If the patient had signs of distant metastases of the lung, bone, intracranial, whole-body bone scan, CT of other parts, and even PET-CT should be added.

10 Clinical evaluations: the judgment of efficacy is mainly based on the following indicators

10.1 The main evaluation indicators

Time to recurrence (TTR): the time between the date of surgery and being found recurrence by examination. In case patients are lost to follow, or with unexplained death, TTR are calculated until this point.

Recurrence: new intrahepatic lesions are found in abdominal CT or MRI with the typical characteristics of enhancement in arterial phase, degradation in portal and delayed phase; chest X-ray finds lung metastasis which should be confirmed by lung CT; If there are any symptoms of adrenal gland, bone, intracranial and other metastasis, abdominal CT, bone scan, and other relative examinations should be used as diagnosis.

10.2 Secondary evaluation indicators

Overall survival (OS): The time between the administrations of the drugs after entering the groups and death or lost follow up of the study individuals.

1, 3, 5-year survival rate: it refers to the percentage of still living individuals after 1 year, 3 years, five-year.

10.3 Tumor markers

According to the results of follow-up, the changes of tumor markers will be evaluated.

10.4 Objective evaluation of efficacy

According to the results of B ultrasound, CT or enhanced MR, chest CT and other imaging studies, recurrence would be assessed.

11. Safety evaluation

From the beginning of that patients signed the informed consent and are selected for trial to one month after the end of treatment, any adverse medical events, regardless of whether a causal relationship with the study medication, will be judged to be Adverse Event (AE).

11.1 Judging criteria of AE and its degree:

We follow NCI-CTC grading criteria (Canada National Cancer Institute with expanding common toxicity criteria by CTC) for AE and its degree judgment. For those adverse reactions that are not listed in the NCI-CTC, we intend to classify the degree as follows:

Mild: patients are easier to accept without induced questions, or patients have only mild discomfort which does not affect their daily lives and there is no need for clinical treatment.

Moderate: patients actively describe the symptoms that affect the life, but they can tolerate, which needs normal clinical treatment.

Severe: patients have objective manifestations, which significantly affect the life, and patients cannot bear to stay in bed, which needs active clinical treatment.

11.2 Records and follow-up of AE

During the test, AE will be accurately recorded, including the time of occurrence, severity,

duration, the measures taken and the outcome. Researchers will follow all the AE until the symptoms of patients disappeared or condition become stable. SAE should be tracked until a proper solution is found even though the study is over.

11.3 Serious adverse event (SAE)

The judgments of SAE:

- Death;
- Life-threatening;
- Leading to hospitalization or prolong hospitalization time;
- Permanent or severe disability.

SAE report system:

The person in charge in the unit and the hospital ethics committee should be reported and SAE report form should be filled in within 24 hours by phone no matter whether any kinds of SAE are related with the drug in 30 days after the treatment. And the form should be reported to national drug administration in time by the person in charge. SAE should be promptly handled, closely tracked until it is properly solved.

Contact method:

1) Contact persons: XIANG GuoAn 13138631120   CHEN KaiYun 13903061359

2) SFDA Safety Supervision

12. Quality assurance

In order to ensure that the experiment could be carried out in strict accordance with the clinical research program, the whole process of clinical trials should be strictly in accordance with requirements of Good Clinical Practice (GCP) issued by National SFDA, which need the researchers ensure the right of patients with standardized test procedures, accurate data, reliable results and credible conclusions. The specific requirements are as follows:

12.1 Requirements of researchers

- Medical staffs who participated in the trial should be organized to systematically study and be seriously familiar with the research program before the study.
- To make sure that all the study procedures are carried out in accordance with the unified program;
- Researchers should talk to the enrolled patients or their designated agents before they signed "informed consent";
- The study should be carried out in strict accordance with the uniform requirements, and Case Report Form (CRF) should be filled in seriously;
- Clinical inspectors should regularly have follow-up;
- Clinical records, the original medical records of the subjects and laboratory records should be completely reserved.

12.2 Requirements of inspectors

Specialized inspectors are selected in order to be in accordance with GCP requirements issued by SFDA in the trial.

- The inspectors should check the subjects' informed consents and situation of screening regularly in the process;
- All CRF should be confirmed to filled in correctly and were consistent with the original data;
- All errors or omissions had been corrected or annotated, which were dated and signed by the

researchers;

- Each subject should be identified and recorded;
- Verification of the exit and lost of the selected subjects should be described in CRF;
- To make sure that all adverse events had been documented, serious adverse events had been reported and recorded;
- Test supplies should be confirmed whether they were supplied, stored, distributed and recovered in accordance with the relevant provisions and appropriate record should be made;
- Each selected patient must complete CRF. The completed original CRF belonged to Second People's Hospital in Guangdong Province, which should not be provided to third parties in any form without the written consent, unless national SFDA.

13. Data analysis and retention

13.1 Case Report Form (CRF)

CRF should be filled in promptly with accuracy by the researchers. CRF should not be altered generally. If there is an error that is needed to be modified, you should sign the place that is modified (CRF filling instructions). CRF is triplicate. After the completed reports are reviewed by Clinical Research Associate (CRA), data are recorded and uploaded. At the same time, the content of CRF can not be modified.

13.2 Statistical analysis

Professional statistical workers will undertake statistical analysis tasks, and participated in the whole process from experimental design and implementation to analysis and summarization. After the completion of the trial protocol and CRF, the development of statistical analysis plan is formulated, and the statistical analysis report will be provided after the necessary changes in the analysis during the testing process.

Statistical methods: the statistical quantitative index includ mean, standard deviation, median, minimum, maximum. All statistical tests use two-sided test, $P <= 0.05$ is considered to have statistically significant difference. The comparison among enumeration data use X2 test. The inter-group comparison of measurement data took use of t test after normal step test and homogeneity test for variance. We use life table for the estimation of survival rate, Log-rank test for the comparison of survival rate among groups and Kaplan-Meier method for survival curves. COX regression analysis will be for the relevant factors affecting patients' survival.

13.3 Data recording and saving

Case Report Form (CRF) use carbonless copy paper, in triplicate. It is required that black pen is used to fill with clean, neat handwriting. CRF should be filled in promptly with accuracy by the researchers. All the contents of CRF should have no omissions or empty (underline non-recorded spaces). It is not permitted to be altered when there is an error, which should be ruled out by single-line with correct parts, date of changing and signature beside. All the data of CRF should be checked with the receivers' records data to ensure there are no errors. For those data that is significantly higher or beyond the clinical acceptation need to be verified. Laboratory sheet copies should stick in laboratory sheet paste of CRF. CRF should be reviewed and sighed for confirmation by principal investigators. After the inspection of complete CRF by CRA, data will be recorded, meanwhile the content of CRF may not be modified.

In order to ensure strictly implementation of relevant provisions of Good Clinical Practice (GCP) issued by SFDA, all the research centers should keep the original records properly, including the

medical records of all subjects, signed informed consent, copies of CRF and the particle distribution records and so on. The retention time is five years after the end of the trial.

14. Ethical principles

14.1 Patients' informed consent

Before each patient is selected into the study, the physician of the study have the responsibility to tell patients or their designated agents the purpose, process, completion and comprehensively introduce seed's properties, possible adverse reactions, risks to bear, possible benefits and other information all-round and detailed, making the patients know their rights. We are ordered to inform the patients of having the right to decide whether to participate in the study, as well as the right to withdraw the study at any time without any discrimination. The patients or their legal representatives should sign the informed consent after carefully reading and fully understanding, and retain the signature page of the copy.

15. Period of the study and participating centers

15.1 Period of the study

July 2000 - June 2004

Starting Date: July 2000

Ending date: June 2004 (follow-up date was 5 years after the surgery)

15.2 Research Center and principal investigators

Affiliations: Second People's Hospital in Guangdong Province, Shanghai Eastern Hepatobiliary Surgery Hospital

Chief person in-charge of the study: Director XIANG GuoAn, Professor SHEN Feng

The clinical study conclusion is based on the statistical analysis of the research data and the results of more than 5 years follow-up, which finally become the clinical study conclusion report.

The report is needed to be reserved in accordance with the provisions after completion.

The relevant data of the study can be published with the agreement of the researchers in responsibility in the study unit.

Annex 1

Implantation of radioactive seeds Iodine-125 for anti-recurrence after radical resection of hepatocellular carcinoma: A prospective randomized control trial

INFORMED CONSENT FORM

Investigator’s Name:___________________________

Investigator’s Address:___________________________

Screening Number: _________________________

Subject Code: _________________________

Doctor’S name：_________________________

Date:

Ⅰ.INTRODUCTION

You have been informed by your doctor that you are suffering from hepatocellular carcinoma （HCC）. As to this kind of disease, the internationally recognized treatment is radical resection .

You are invited to participate in the research project by Dr. __________ : to evaluate the inhibitory effect of adjuvant iodine-125 (^{125}I) brachytherapy on postoperative recurrence of HCC in our study..

As a research subject of this study, you have the right to know the consequences of participating in this study. The following information helps you to understand the possible benefits and risks of participating in the study. After that, you can make a decision of whether or not to participate in it. Your participation in this study is strictly voluntary and you have no obligation whatsoever. You may agree or refuse to participate in the study.

The Ethics Committee of this hospital is responsible for ensuring the rights, safety and well-being of research subjects. This study has received approval from the Ethics Committee of this hospital. If you decide to participate, you will be enrolled for the study and will remain in the study until:

1. You no longer consent to participate in the study.
2. Your doctor informs you that the study is terminated.

If you are not able to understand any part of this document or if you have any questions, please ask your doctor before signing the attached consent form.

It is important that you read this document thoroughly and discuss it with your doctor and anyone else you prefer before signing the attached consent form. Your signature on this consent form (with date) will be required before the doctor can thoroughly assess whether or not you are eligible to participate in this study.

Ⅱ.PURPOSE

This study will examine whether adjuvant ^{125}I brachytherapy for HCC patients after resection could reduce postoperative tumor recurrence rate and increase overall survival (OS) rate as well as adverse reactions of this treatment. Our primary testing indicators are tumor recurrence rate in five years，OS rate in one, three or five years; the condition of hepatic function of patients after surgery, the incidence rate of complications (mainly about hepatic failure) and the decline level of serum AFP concentration.

Ⅲ.RANDOMIZATION

Whether adjuvant ^{125}I brachytherapy for HCC patients after resection could reduce

postoperative tumor recurrence rate is not unclear. The most unbiased way to decide which treatment to be given to you will be via randomization which is like tossing a coin. There are equal chances of you being allocated to either treatment. Neither you nor your doctor will be allowed to decide which treatment you are allocated to.

Ⅳ.**<u>TREATMENT</u>**

If you agree to participate and go through the proper process of randomization, you will receive either of the following treatments, as informed by your doctor.

Group A: The adjuvant ^{125}I brachytherapy group
Based on curative resection of liver tumor, ^{125}I seed implantation will be applied.
Group B: Control group
You will received curative resection of liver tumor. The rest treatment was the same as that of the ^{125}I treated group except for implantation of ^{125}I.

Ⅴ.**<u>Procedure of Research</u>**

This study is being conducted in our hospital, and it will involve 150 pairs' cases viz. 300 patients.

If you decided to participate in this study, your doctor will determine whether you are eligible for the trial according to the criteria set forth in the protocol.

You will receive the treatment that is allocated to you. After the treatment ends， your doctor will continuously follow your condition every month if not otherwise informed by the doctor.

If you participate in this study, your doctor will perform the following procedures:

- Inquire about your medical history (previous/current illness, concomitant illness, allergies etc.)-recorded within 7 days prior to randomization.
- Inquire about your current status –within 7days prior to randomization and at every following-up.
- Perform physical examination-within 7 days prior to randomization and at every following-up.
- Invite you to complete a Quality of Life questionnaire-within 7 days prior to randomization, every 6 weeks during treatment, and at every following-up.
- Perform Electrocardiogram-within 7 days prior to randomization and whenever deemed necessary by your doctor.
- Perform chest X-ray-performed within 7days and whenever deemed necessary by your doctor.
- Perform MRI or CT scan –performed within 7 days prior to randomization and whenever deemed necessary by your doctor (usually every 8-12weeks during the

follow-up period).

- Perform Laboratory tests –A small sample of blood will be taken from a vein in the arm. The blood tests will be performed within 7 days prior to randomization and at every following-up.
- Perform pregnancy test –for women of childbearing potential, this will be performed within 7days prior to randomization and whenever deemed necessary by your doctor

Ⅵ.**<u>THINGS TO NOTE</u>**

Whichever treatment you are allocated to, please take note of the following:

- In most cases, procedures will only be performed according to the frequency mentioned above. However, your doctor may decide to perform extra procedures as he deems necessary.
- If you participate in this study, please follow the treatment procedures as advised by your doctor. If you do not feel well or experience any side effects, please inform your doctor immediately.
- Your doctor will keep in contact with you.
- If you participate in this study, you have to inform your doctor the following information: the medications you are taking, if you are seeing another doctor, if you received any new treatment, if you take part in another research study, if you feel any different since the last time you saw your doctor.
- If you are invited to take part in other research studies, please let them know you are participating in this study.

Ⅶ. **<u>PREGNANCY/CONTRACEPTION</u>**

At this moment, it is still unknown what kind of effects the study treatment will have on foetus. Females of child-bearing potential and males with partners of childbearing potential must use medically appropriate contraception (to be decided with your doctor).

Ⅷ. **<u>RISKS</u>**

There are certain risks and discomforts that may be associated with any research. It is the same with this study. You may experience slight pain or bruising during the drawing of blood for laboratory tests. There may occur some complications and unexpected incidents while surgical treatment, as well as transcatheter arterial chemoembolization are performed. The following are the complications and unexpected incidents found in the previous studies:

Surgical treatment:

The common complications or adverse reactions during an operation or after include: 1. bleeding, haemorrhea, or even haemorrhagic shock; 2. blunt abdominal injury (BAI) during the operation; 3. cardiovascular accidents; 4. stress ulcer in disgestive tract, bleeding, or even perforation; 5. hepatic failure; 6. multiple organ failure (renal failure, respiratory failure, or heart failure); 7. bile leakage; 8. intestinal adhesion or intestinal obstruction; 9. bad healing of incision or failure to heal; 10. recurrence or metastasis of a tumor.

Implantation ^{125}I seeds

There is no related serious adverse reactions were reported in treatment with iodine 125 seeds.Nevertheless, in the study may not predict adverse reactions occurs, but the doctor will closely observe the changes in your condition, make every effort to ensure the safety.

CONCLUSION

You may experience one, several or none of the complications or unexpected incidents. It may be mild, intermediate or severe effects. Besides, during the participation period, some rare or potential unknown risks may occur. Once any risk occurs, including the above-mentioned slight illnesses, you shall inform your doctor and he will provide immediate and adequate medical care to alleviate your discomfort. During the course of the study, if there is any important new information available regarding whether you will continue to participate in the study, you will be informed on a timely basis.

Ⅸ. **BENEFITS**

The data collected from this study will help doctors to know how to better manage and treat patients like you. This will be beneficial for patients in the future.

During the study, you will receive better medical treatment, which will make your tumor curing or become smaller or keep it in check. Therefore, your life may be prolonged and the quality of life will be improved. However, the therapy may also turn out to give you no benefits.

Ⅹ. **CONFIDEDTIALITY**

Your privacy shall be protected. The information obtained during this study, including your medical records, will be kept strictly confidential according to applicable law and regulations.

Your doctor will collect your data, including study records, your overall health status, your response to the treatment, the side effects you experienced, all treatments and laboratory results obtained during the study. All your information will be identified by a code number and initials, and your identity will be kept confidential.

Your data may be analyzed, and the results may be presented at international congresses or published in international journals. This is to allow other doctors to understand the results of the trial. You will not be identified in any research publications including journal articles, papers, and/or research presentations. If you withdraw from the study, the information collected from you will still be included in the analysis.

During the study and up to 15 years after completion of the study, qualified representatives of the sponsor, the Institutional Review Board (IRB)/Ethics Committee (EC), and/or domestic regulatory authorities, according to local regulations, may review your medical records in order to determine the accuracy of the reported data and/or to protect your safety and welfare. Records that reveal your identity will be kept confidential by the people who review them.

Ⅺ. STUDY COSTS/COMPENSATION

We will not pay for expenses of your routine test and treatment or any other procedures not specifically required by the study protocol.

We shall not be responsible for events unrelated to the study for which you may encounter. We will not be liable for any expenses incurred during the management and treatment of expected or possible toxicity, for example, complications of surgery，complications of endoscopic setting of stents.

Ⅻ. ADDITIONAL INFORMATION

You are encouraged to put forward questions at any time of the experimental period. If you have any questions regarding the study, procedure, benefits, the risks or something while on treatment, you may contact your study doctor:

Dr：______________________________

Tel No.:__________________________

XIII.PARTICIPATION/STUDY WITHDRAWAL

Your participation is strictly voluntary and you may refuse to participate and/or withdraw consent and discontinue participation in the project at any time without penalty or loss of benefits to which you are otherwise entitled.

Your participation in this study may be discontinued without your consent by your doctor or by sanofi-aventis if, in the doctor`s and/or sponsor`s opinion, it is in your best interest to discontinue the study.

Should you choose to discontinue your treatment, additional information may continue to be obtained from your medical records for the purpose of study following-up.

We may choose to permanently terminate the study which will not need your consent in advance. In this case, you will be informed by your doctor. He/she will discuss further treatment with you.

INFORMED CONSENT

I hereby declare that I have read the above patient`s information sheet for the study entitled: ____

____

●I have been well-informed about the purpose, set-up, course, foreseeable benefits and risks of the study. At any time during and after my participation to the study, I am aware the investigator is responsible for providing me any additional information about the study as well as in case of a study related injury.

●I am aware that my participation is voluntary and that I can withdraw from the study at any time with no negative consequences.

●In view of the requirements of the study, I agree that data arising from the study, including data on my health status will be collected by the doctor during this study. I accept that the research data from the study will be handed over confidentially by the investigating center, to personnel involved in the study, as designated by the Sponsor, and to the Health and Regulatory Authorities. I agree that the sponsor and its representatives will be granted direct access to original medical records to verify clinical trial procedures and/or data, which will be handled in a confidential manner. Even if I withdraw from the study, I agree to the use of the data that had already been collected to be analyzed for the study.

●I agree to participate in the study. I am aware that the study treatments are experimental and there are associated risks.

●My name or any material identifying me as a study participant will not be released without my written permission, except in case as such release is required by law.

I declare that I have truthfully answered all questions about my medical past and that I will follow all rules and regulations imposed upon me by the research staff and listed on the patient`s information sheet.

After signing, I will receive a copy of the written informed consent.

Name of patient Signature Data

____ __________

I have given the subjects enough time to understand the subject instruction, informed consent form, and experiment protocol, and tried my best to answer them all.

Name of doctor Signature Date
